# Supplementary material for: Structure and function of a family of tick-derived complement inhibitors targeting properdin
Source: Nat Commun. 2022 Jan 14;13:317. doi: 10.1038/s41467-021-27920-2 (PMC8760278; doi:10.1038/s41467-021-27920-2)
Supplement: Supplementary file 1 — Supplementary Information [file 41467_2021_27920_MOESM1_ESM.pdf]

Supplementary Information For:

Targeting properdin - Structure and function of a family of tick-derived complement inhibitors targeting properdin

Katharina Braunger*[,1], Jiyoon Ahn*[,1], Matthijs M. Jore*[,1,5], Steven Johnson[1,2], Terence T. L. Tang [1,6], Dennis V. Pedersen [3], Gregers R. Andersen[3] and Susan M. Lea [1,2,4]

*These authors contributed equally

[1] Sir William Dunn School of Pathology, University of Oxford, OX1 3RE Oxford, United Kingdom

[2] Center for Structural Biology, Center for Cancer Research, National Cancer Institute, Frederick, MD 21702, United States of America

[3] Department of Molecular Biology and Genetics, Aarhus University, DK-8000 Aarhus, Denmark

[4] Central Oxford Structural Molecular Imaging Centre, University of Oxford, OX1 3RE Oxford, United Kingdom

[5] present address: Department of Medical Microbiology, Radboud University Medical Centre, Nijmegen, Netherlands

[6] present address: MRC Laboratory of Molecular Biology, Cambridge, United Kingdom

Correspondence should be addressed to S.M.L. (susan.lea@nih.gov) or S.J. (steven.johnson2@nih.gov)

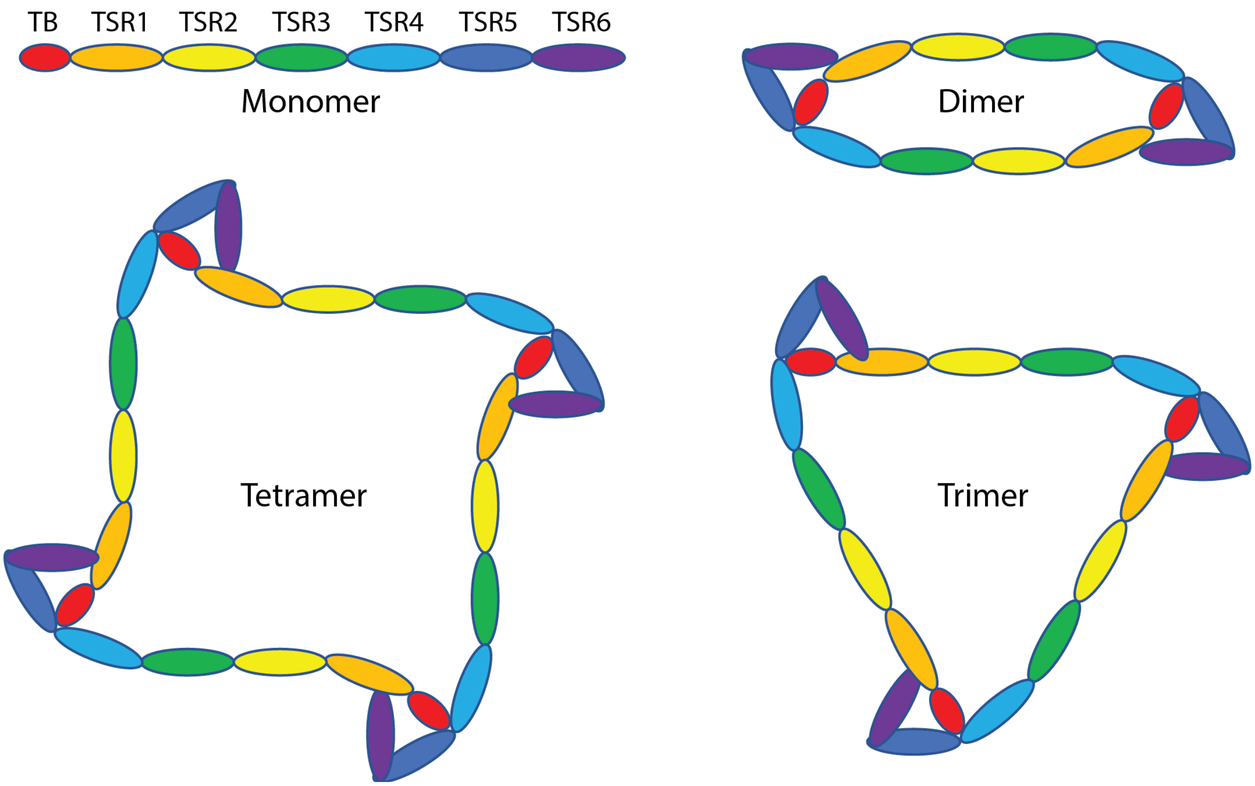

Supplementary figure 1. Properdin oligomeric states.
Schematic representation of the major properdin oligomeric states, as predicted by the crystal structures of monomerized properdin. Domains are color-coded as described for the monomer.

a) MonoQ fractionation b) SEC fractionation

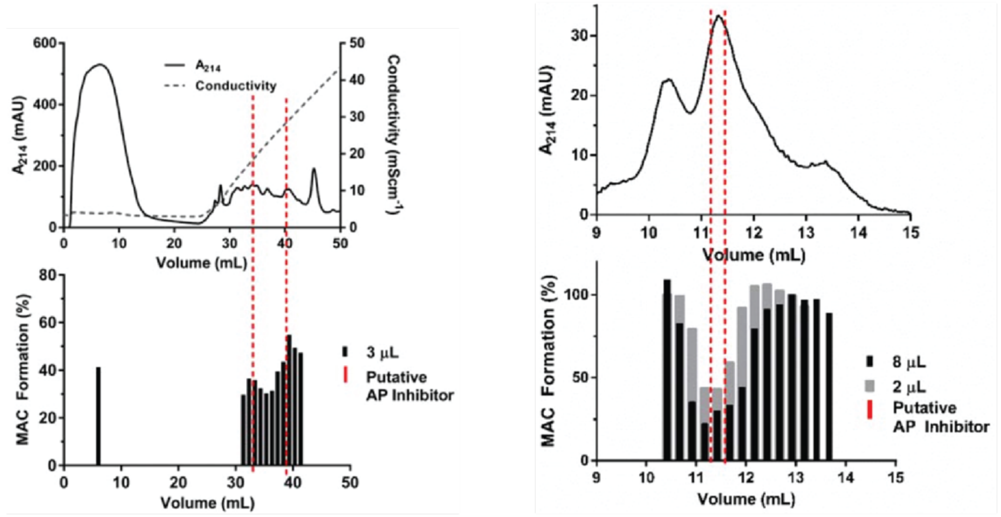

c)

Accession No.	Re-name	Score	Mature Protein Mass (Da)	pI	BLAST Top Hit	BLAST E score	BLAST RefSeq
comp52_seq0	AP1	239	20068	4.67	None		
comp1106_seq1	AP2	79	19433	5.97	Salivary lipocalin (A. variegatum)	1.00E-13	DAA34698.1
comp3626_seq0	AP3	130	45968	5.16	Hypothetical Protein, IscW, Ixodes scapularis	0	XP_002409462.1
comp1215_seq0	AP4	110	38706	6.15	Proliferation-associated Protein 2G4	1.00E-157	KDR23244.1
comp8435_seq1	AP5	81	22430	4.89	None		
comp5629_seq0	AP6	71	24630	8.79	None		
comp4_seq0	AP7	36	21995	5.38	Lipocalin	5.00E-4	ABI52661.1
RpIx75-921729	-	612	22957	5.21	Salivary lipocalin (A. variegatum)	3.00E-11	DAA34698.1
RpSigP-58530	-	24	21995	5.38	N(G),N(G)-di-methylarginine dimethylamino-hydrolase	3	WP_011213937.1

d)

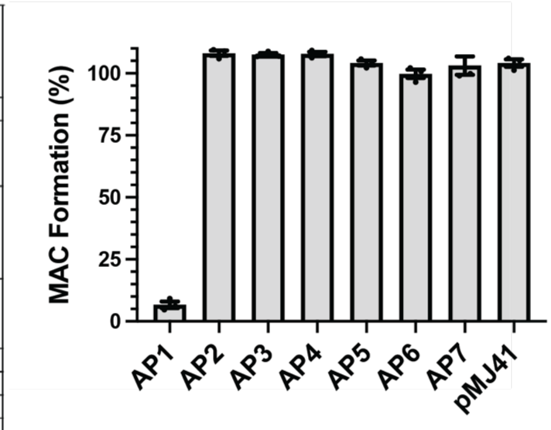

Supplementary figure 2. Fractionation of alternative pathway inhibitory activity.
(a) Salivary gland extract from 250 pairs of salivary glands, diluted with 2mM Na_2HPO_4/NaH_2PO_4, pH 7.0, was applied to a MonoQ 5/50 GL column (GE) and eluted with a 0-0.5 M NaCl gradient. Complement inhibitory fractions were pooled and further purified by HIC (Fig. 1b). (b) Size exclusion chromatography of the complement inhibitory fraction from HIC. Sample was run on a Superdex75 10/300 column (GE). (c) Seven protein sequences identified by mass spectrometry from the SEC peak were analyzed by BLAST. (d) Supernatants from S2 cell cultures expressing the seven hits identified by MS (plus an empty vector control) were used in a complement inhibition ELISA assay. Error bars are Mean with s.e.m. for three technical replicates (n=3). Only AP1 demonstrated complement inhibition activity.

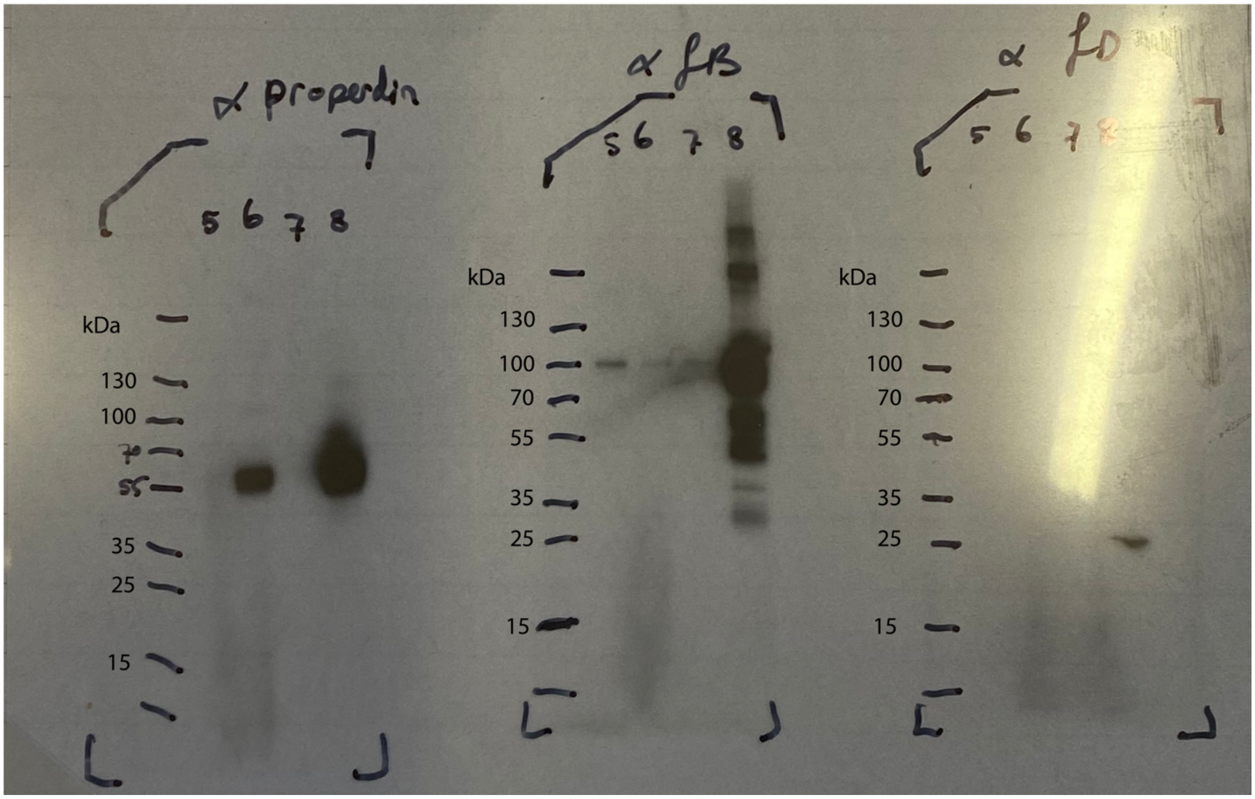

Supplementary figure 3. Alternative complement pathway pull-down analysis.
Purified CirpA1 was covalently coupled to NHS-activated magnetic beads (ThermoFisher) and incubated with 50 μl human serum, followed by removal of excess serum and washing in PBS. Beads were run on SDS-PAGE and proteins visualized on Western blot using anti-properdin, anti-Factor B or anti-Factor D antibodies. Lane 5=Empty bead control. Lane 6=CirpA1 coated beads. Lane 7=Salp20 coated beads. Lane 8=Purified properdin, Factor B, Factor D mix. Representative result of n=2.

a)

	CirpA1	CirpA2	CirpA3	CirpA4	CirpA5	CirpA6	Organism of origin
CirpA1	100	82	52	48	46	43	*Rhipicephalus pulchellus*
CirpA2	82	100	72	57	48	44	*Rhipicephalus pulchellus (m, IH)*
CirpA3	52	72	100	56	42	47	*Rhipicephalus pulchellus (f, IH)*
CirpA4	48	57	56	100	42	44	*Rhipicephalus appendiculatus*
CirpA5	46	48	42	42	100	44	*Rhipicephalus appendiculatus (IH)*
CirpA6	43	44	47	44	44	100	*Rhipicephalus microplus*

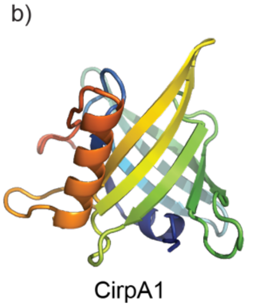

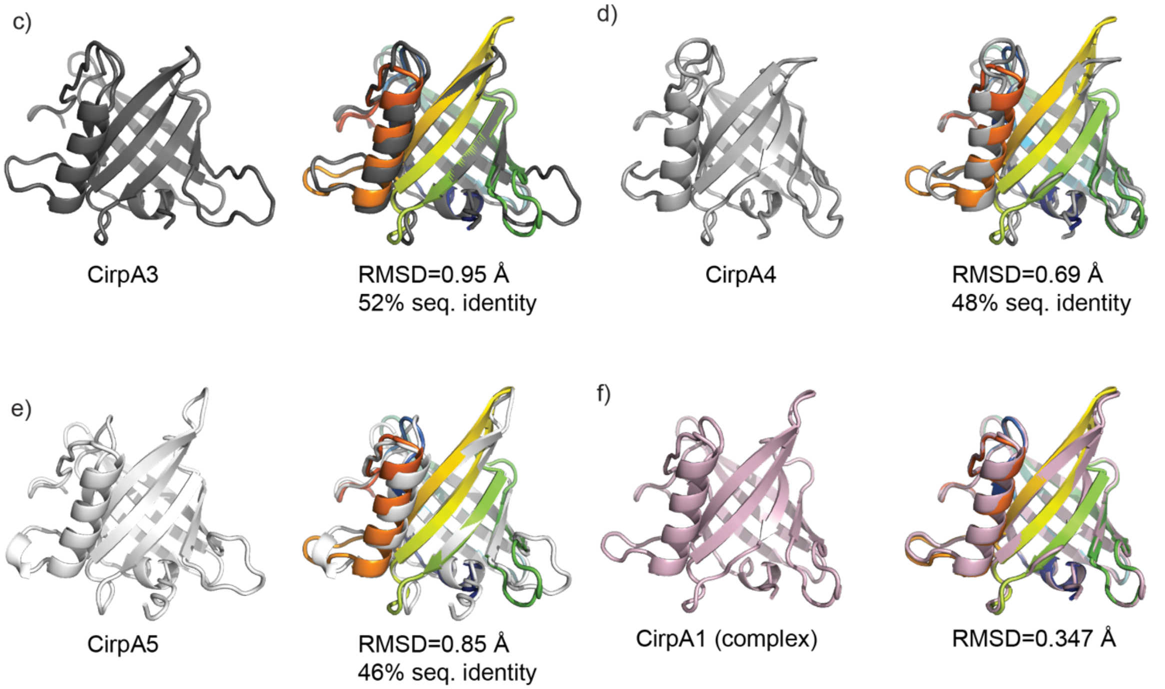

Supplementary figure 4. Sequence and structure analysis of CirpA homologues.
(a) Pairwise sequence identity of the CirpA family. (b) Structure of CirpA1 colored from N (blue) to C (red) terminus. (c) Structure of CirpA3 (grey) overlayed with CirpA1 (rainbow). (d) Structure of CirpA4 (grey) overlayed with CirpA1 (rainbow). (e) Structure of CirpA5 (grey) overlayed with CirpA1 (rainbow). (f) Structure of CirpA1 (pink) from the CirpA:Properdin complex overlayed with free CirpA1 (rainbow).

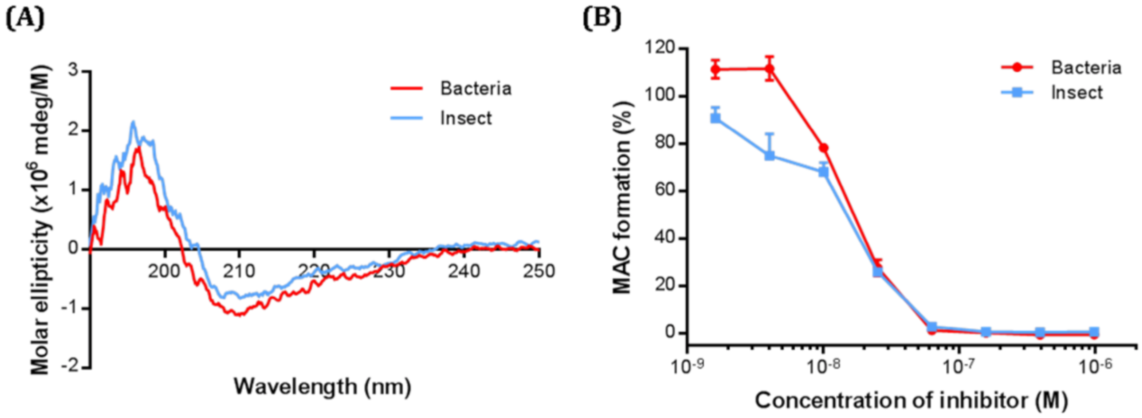

Supplementary figure 5. Comparison of CirpA1 produced from insect and bacterial cells.
(A) Circular dichroism spectroscopy of CirpA1 produced from S2 cells (blue) and refolded from *E. coli* inclusion bodies (red). (B) Alternative pathway inhibition of CirpA1 produced from S2 cells (blue) and refolded from *E. coli* inclusion bodies (red). Error bars are Mean with s.e.m. for three technical replicates.

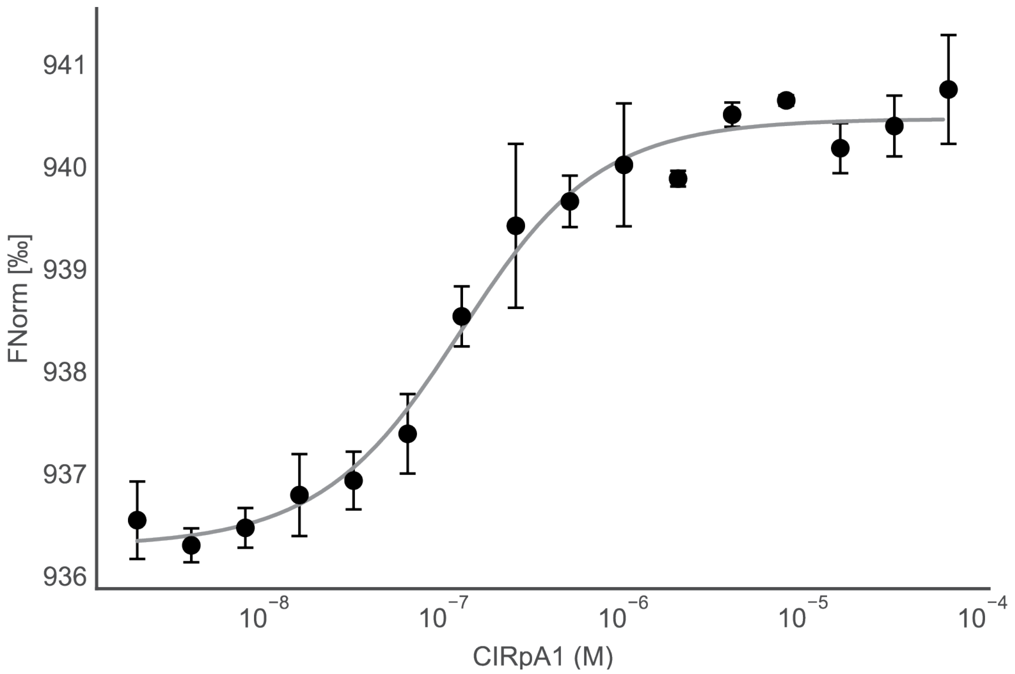

Supplementary figure 6. Micro-scale thermophoresis of the CirpA1:Properdin interaction. Labelled, FPΔ2,3 (50 nM) was incubated for 5 min with unlabeled CirpA1 at concentrations between 2 nM and 64 μM. MST measurements were performed on a Monolith NT.115 at 20% LED power and low MST power. An MST on time of 2.5 s was used for analysis via the MO.Affinity Analysis 2.3 Software, and a K_d of 97 nM +/- 18 nM was derived for the interactions (n=3 independent measurements, error bars represent the standard deviation of the mean).
