# Supplementary material for: Structure and function of a family of tick-derived complement inhibitors targeting properdin
Source: Nat Commun. 2022 Jan 14;13:317. doi: 10.1038/s41467-021-27920-2 (PMC8760278; doi:10.1038/s41467-021-27920-2)
Supplement: Supplementary file 3 — Reporting Summary [file 41467_2021_27920_MOESM3_ESM.pdf]

nature research

Corresponding author(s): SUSAN LEA, STEVEN JOHNSON

Last updated by author(s): Nov 17, 2021

Reporting Summary

Nature Research wishes to improve the reproducibility of the work that we publish. This form provides structure for consistency and transparency in reporting. For further information on Nature Research policies, see our Editorial Policies and the Editorial Policy Checklist.

Statistics

For all statistical analyses, confirm that the following items are present in the figure legend, table legend, main text, or Methods section.

n/a	Confirmed	
☐	☒	The exact sample size (*n*) for each experimental group/condition, given as a discrete number and unit of measurement
☐	☒	A statement on whether measurements were taken from distinct samples or whether the same sample was measured repeatedly
☐	☒	The statistical test(s) used AND whether they are one- or two-sided<br>*Only common tests should be described solely by name; describe more complex techniques in the Methods section.*
☒	☐	A description of all covariates tested
☐	☒	A description of any assumptions or corrections, such as tests of normality and adjustment for multiple comparisons
☐	☒	A full description of the statistical parameters including central tendency (e.g. means) or other basic estimates (e.g. regression coefficient) AND variation (e.g. standard deviation) or associated estimates of uncertainty (e.g. confidence intervals)
☐	☒	For null hypothesis testing, the test statistic (e.g. *F*, *t*, *r*) with confidence intervals, effect sizes, degrees of freedom and *P* value noted<br>*Give P values as exact values whenever suitable.*
☒	☐	For Bayesian analysis, information on the choice of priors and Markov chain Monte Carlo settings
☒	☐	For hierarchical and complex designs, identification of the appropriate level for tests and full reporting of outcomes
☒	☐	Estimates of effect sizes (e.g. Cohen's *d*, Pearson's *r*), indicating how they were calculated

Our web collection on statistics for biologists contains articles on many of the points above.

Software and code

Policy information about availability of computer code

Data collection	Data was processed using Xia2 (version 0.5.542) or STARANISO (version 2.2.19).
Data analysis	X-ray diffraction data were analysed using the CCP4 (version 7.0, Acta Crystallographica Section D, 67, 235-242 (2011))and Phenix (version 1.18.2, Acta Crystallographica Section D: 75, 861-877 (2019)) suites of programs. COOT (version 0.9) was used for model building Electrostatics were calculated using APBS (version 3.0, Protein Science, 27 112-128 {2018)) within Pymol v2.4 (Schrodinger). Assays were analysed using GraphPad Prism 7 {GraphPad LLC).

For manuscripts utilizing custom algorithms or software that are central to the research but not yet described in published literature, software must be made available to editors and reviewers. We strongly encourage code deposition in a community repository (e.g. GitHub). See the Nature Research guidelines for submitting code & software for further information.

Data

Policy information about availability of data

All manuscripts must include a data availability statement. This statement should provide the following information, where applicable:

- Accession codes, unique identifiers, or web links for publicly available datasets
- A list of figures that have associated raw data
- A description of any restrictions on data availability

X-ray coordinates and data have been deposited into the Protein Data Bank (PDB), www.pdb.org under accession codes PDB 7B2D (CirpA1), PDB 7B28 (CirpA3), PDB 7B29 (CirpA4), PDB 7B2A (CirpA5), PDB 7B26 (CirpA1-FPDTSR2,3). CirpA inhibitor sequences have been deposited into the GenBank under accession codes MW260265 (CirpA1), MW260267 (CirpA2) and MW260266 (CirpA3); or have already been publicly available under accession codes CD794868 (CirpA4), GEFJ01011401 (CirpA5) an CK182034 (CirpA6).

April 2020

Field-specific reporting

Please select the one below that is the best fit for your research. If you are not sure, read the appropriate sections before making your selection.

- [x] Life sciences
- [ ] Behavioural & social sciences
- [ ] Ecological, evolutionary & environmental sciences

For a reference copy of the document with all sections, see nature.com/documents/nr-reporting-summary-flat.pdf

Life sciences study design

All studies must disclose on these points even when the disclosure is negative.

Sample size	Sample sizes were not precalculated, but were determined to ensure the maximum number of truly independent repeats from the material reasonably available.
Data exclusions	No data were excluded
Replication	All biochemical assays contain replicates as described in the methods and figure legends
Randomization	Not applicable. Samples were directly compared, i.e. WT vs mutant vs control.
Blinding	Not applicable as subjective analysis of data was not performed.

Reporting for specific materials, systems and methods

We require information from authors about some types of materials, experimental systems and methods used in many studies. Here, indicate whether each material, system or method listed is relevant to your study. If you are not sure if a list item applies to your research, read the appropriate section before selecting a response.

Materials & experimental systems

n/a	Involved in the study	
[ ]	[x]	Antibodies
[ ]	[x]	Eukaryotic cell lines
[x]	[ ]	Palaeontology and archaeology
[x]	[ ]	Animals and other organisms
[ ]	[x]	Human research participants
[x]	[ ]	Clinical data
[x]	[ ]	Dual use research of concern

Methods

n/a	Involved in the study	
[x]	[ ]	ChIP-seq
[x]	[ ]	Flow cytometry
[x]	[ ]	MRI-based neuroimaging

Antibodies

Antibodies used	1) Primary antibody (goat α-FP: 1:2,000, Complement Technology, Cat # A239).<br>2) Secondary antibody (donkey α-goat HRP, Promega, 1:10,000, Cat # V8051).<br>3) Penta-His HRP Conjugate Kit(Qiagen, 1:2000, Cat # 34460)<br>4) Anti-sheep red blood cell stroma (Sigma-Aldrich, Cat # S1389)
Validation	1) As detailed in - https://www.complementtech.com/catalog/product/complement-polyclonal-antisera/goat-anti-human-factor-p/<br>2) As detailed in - https://www.promega.com/products/protein-detection/primary-and-secondary-antibodies/donkey-anti-goat-igg-hrp/?catNum=V8051#resources<br>3) As detailed in - https://www.qiagen.com/us/products/discovery-and-translational-research/protein-purification/tagged-protein-expression-purification-detection/anti-his-hrp-conjugate-kits/<br>4) As detailed in - https://www.sigmaaldrich.com/US/en/product/sigma/s1389

Eukaryotic cell lines

Policy information about cell lines

Cell line source(s)	HEK293F cells were purchased from ThermoFisher (Cat # R79007)
Authentication	Authentication was carried out by the manufacturer. See - https://www.thermofisher.com/order/catalog/product/R79007
Mycoplasma contamination	Mycoplasma certification is carried out by the manufacturer.

Commonly misidentified lines (See ICLAC register)	None

Human research participants

Policy information about studies involving human research participants

Population characteristics	Not applicable. Healthy donors provided small volumes of blood that was immediately used to provide serum for reagent purification. Serum from multiple donors was pooled and aliquots frozen.
Recruitment	Voluntary donors were sought from the laboratory. Donor consent was obtained.
Ethics oversight	As all cellular material was immediately destroyed and serum was used as a source of reagents, no ethics approval was required as per Oxford University OHS policy document 1/03.

Note that full information on the approval of the study protocol must also be provided in the manuscript.
